# Autophagy in Adipocyte Browning: Emerging Drug Target for Intervention in Obesity

**DOI:** 10.3389/fphys.2019.00022

**Published:** 2019-01-28

**Authors:** Seung-Hyun Ro, Yura Jang, Jiyoung Bae, Isaac M. Kim, Cameron Schaecher, Zachery D. Shomo

**Affiliations:** ^1^Department of Biochemistry, University of Nebraska–Lincoln, Lincoln, NE, United States; ^2^Department of Neurology, Johns Hopkins University School of Medicine, Baltimore, MD, United States; ^3^Department of Cell and Regenerative Biology, University of Wisconsin School of Medicine and Public Health, Madison, WI, United States; ^4^College of Medicine, University of Nebraska Medical Center, Omaha, NE, United States

**Keywords:** autophagy, lipophagy, mitophagy, beige/brown adipose tissue, browning, white adipose tissue, whitening, obesity

## Abstract

Autophagy, lipophagy, and mitophagy are considered to be the major recycling processes for protein aggregates, excess fat, and damaged mitochondria in adipose tissues in response to nutrient status-associated stress, oxidative stress, and genotoxic stress in the human body. Obesity with increased body weight is often associated with white adipose tissue (WAT) hypertrophy and hyperplasia and/or beige/brown adipose tissue atrophy and aplasia, which significantly contribute to the imbalance in lipid metabolism, adipocytokine secretion, free fatty acid release, and mitochondria function. In recent studies, hyperactive autophagy in WAT was observed in obese and diabetic patients, and inhibition of adipose autophagy through targeted deletion of autophagy genes in mice improved anti-obesity phenotypes. In addition, active mitochondria clearance through activation of autophagy was required for beige/brown fat whitening – that is, conversion to white fat. However, inhibition of autophagy seemed detrimental in hypermetabolic conditions such as hepatic steatosis, atherosclerosis, thermal injury, sepsis, and cachexia through an increase in free fatty acid and glycerol release from WAT. The emerging concept of white fat browning–conversion to beige/brown fat–has been controversial in its anti-obesity effect through facilitation of weight loss and improving metabolic health. Thus, proper regulation of autophagy activity fit to an individual metabolic profile is necessary to ensure balance in adipose tissue metabolism and function, and to further prevent metabolic disorders such as obesity and diabetes. In this review, we summarize the effect of autophagy in adipose tissue browning in the context of obesity prevention and its potential as a promising target for the development of anti-obesity drugs.

## Introduction: Autophagy in Adipocytes

### Autophagy

Macroautophagy, generally referred to as autophagy, is a cytosolic degradation and recycling process of damaged organelles and unwanted components in the cell (Singh et al., 2009b; Zhang et al., 2012; Cairo et al., 2016). When the cells or tissues are limited in their nutrient supply or exposed to a substantial amount of environmental, oxidative, or genotoxic stresses, autophagy as a cellular survival and defense mechanism can be activated (Zhang et al., 2012; Bluher, 2013; Choi et al., 2013; Cairo et al., 2016). Autophagy can be induced in the cell through inhibition of either the nutrient-sensing kinase, mechanistic target of rapamycin complex 1 (mTORC1), or the activating-stress-sensing kinase, 5′-AMP activated protein kinase (AMPK) (Jung et al., 2010; Kim et al., 2011; Stienstra et al., 2014). On the other hand, when cells or tissues are supplied with excessive nutrients, the autophagy process is not necessary and is attenuated through mTORC1 activation and AMPK inhibition (Jung et al., 2010; Kim et al., 2011; Stienstra et al., 2014). When autophagy is suppressed for an extended period of time from continuous overnutrition, as with a high fat and/or fructose diet, the accumulation of unwanted proteins and organelles in the major metabolic tissues – such as adipose, liver, muscle, and pancreas – can become detrimental and eventually induce metabolic dysfunction and diseases such as obesity and diabetes (Zhang et al., 2012; Bluher, 2013; Rocchi and He, 2015). However, contradictory to findings of previous studies, autophagy seems hyperactivated in an effort to generate more fats from recycled energy in adipose tissues of obese patients (Kovsan et al., 2011; Jansen et al., 2012). A few reports suggest that autophagy inhibition can be a protective mechanism against high-fat diet-induced metabolic dysfunction by converting white adipose tissue (WAT) to brown adipose tissue (BAT) (Armani et al., 2014; Parray and Yun, 2017). Throughout the last decade, the therapeutics of modulating autophagy activity have drawn much attention; however, their clinical effectiveness in improving the metabolic profiles of humans with adipocyte metabolic dysfunctions linked to overweight, obesity, and diabetes has not been ascertained (Shoji-Kawata et al., 2013; Galluzzi et al., 2017).

### Adipogenesis

Adipogenesis is a unique adipocyte differentiation process that generates lipid droplets with triglycerides and fatty acids inside the lipid vacuoles (Rosen and Spiegelman, 2006; Ro et al., 2013; Ahmed et al., 2018). Autophagy for non-selective bulk degradation of proteins and lipids through the fusion of autophagosomes and lysosomes is suggested as one of the major types of autophagy in adipocytes (Singh et al., 2009b; Singh and Cuervo, 2012; Ro et al., 2013). The relationship between adipogenesis and autophagy has drawn much attention over the past decade regarding the potential link to metabolic diseases such as obesity. Autophagy is necessary and activated when white adipocyte undergoes differentiation (Singh et al., 2009b; Singh and Cuervo, 2012; Zhang et al., 2012; Ahmed et al., 2018). Chloroquine treatment and autophagy-related protein (ATG) 5 knockdown decreases adipogenesis of mouse embryonic fibroblast (MEF) cells. Targeted deletion of ATG5 in mice leads to a dramatically reduced mass of Perilipin A-positive white adipocytes in late-stage embryos and neonatal pups (Baerga et al., 2009; Singh et al., 2009b). Singh et al. and other research groups also observed decreased levels of microtubule-associated protein 1A/1B-light chain 3 (LC3), peroxisome proliferator-activated receptor (PPAR)- γ, and triglyceride. This finding indicates that adipocyte differentiation and lipid accumulation are blocked by inhibition of autophagy when ATG7 is knocked down in adipocytes or in adipose-specific deletion in mice (Singh et al., 2009b; Zhang et al., 2009; Singh and Cuervo, 2012). When ULK1, the mammalian homolog of ATG1 and the downstream autophagy kinase target of mTORC1, is knocked down in 3T3-L1 white adipocytes, adipogenesis increases, although autophagy is inhibited. However, the ULK2 knockdown in white adipocytes blocks both autophagy and adipogenesis (Ro et al., 2013). An increase in autophagy has been reported in adipose tissues derived from obese humans and mice, supporting adipogenesis in forming and storing more fat depots in the face of overnutrition (Kovsan et al., 2011; Cummins et al., 2014; Kosacka et al., 2015). Both an increased level of the lipidated form of LC3 (LC3-II) as an autophagosome marker and a decreased level of ubiquitin-binding scaffold protein p62, also called sequestosome 1(SQSTM1), were observed in obese humans and mice, the combination of which would appear to be consistent with an increase in autophagy activity during adipogenesis (Klionsky et al., 2016; Yoshii and Mizushima, 2017). The level of autophagy activity seems to differ or at least to fluctuate depending on the adipose tissue type, external stimuli, and tissue age (Bluher, 2013; Kosacka et al., 2015; Li and Ding, 2017). Autophagy is activated when white adipocyte is undergoing differentiation to form lipid droplets. However, in an opposite way, autophagy also can be inhibited when brown adipocyte is activated with UCP1 and PPAR-γ, increasing thermogenesis and browning, respectively, under cold exposure (Singh and Cuervo, 2012; Cairo et al., 2016; Ferhat et al., 2018). Based on previous studies on autophagy in different types of adipocytes, the inhibition of autophagy seems to be better for obesity prevention by reducing the formation of lipid droplets in white adipocytes and promoting energy expenditure in beige or brown adipocytes (Singh et al., 2009b; Kovsan et al., 2011; Ro et al., 2013; Cairo et al., 2016).

### Adipocytes

Adipocytes are the main type of cells found in both white and brown adipose tissues (Lo and Sun, 2013). White adipocytes contain a single lipid droplet and a small number of mitochondria. Brown adipocytes contain multiple small lipid droplets, enriched amounts of mitochondria, and exhibit a unique thermogenesis function through uncoupling protein 1 (UCP1). Beige adipocytes are the brown-like cells located within WAT, and they have a higher expression level of UCP1 than white adipocytes (Wu et al., 2012; Lo and Sun, 2013; Armani et al., 2014; Cummins et al., 2014). Browning is a process of dynamic conversion or modification of white adipocytes into beige/brown adipocytes upon activation by exposure to physiological, pharmacological, or hormonal stimuli (Wu et al., 2013; Abdullahi and Jeschke, 2016). Browning of white adipocytes is generally induced under cold exposure and exercise (Wu et al., 2012; Lo and Sun, 2013; Aldiss et al., 2018). However, this process does not completely transform or transdifferentiate white adipocytes into brown adipocytes. The white adipocytes become only a brown adipocyte-like phenotype, which is also called beige, inducible brown, brown-in-white, or brite adipocyte (Bartelt and Heeren, 2014; Scheele and Nielsen, 2017). In mouse studies, more beige cells have been detected in WAT of lean, compared to obese mice (Rachid et al., 2015). However in human studies, they only observed higher native BAT activity but not active beige cells in lean subjects (Vijgen et al., 2011). Conventional methods to increase UCP1 in beige/brown fat cells, such as cold acclimation in humans, have not revealed any inducible browning fat depots in addition to the constitutively present depots and have not also proven enough to mediate browning of white fat depots (Scheele and Nielsen, 2017). There indeed are a few reports on cold-induced browning of human perirenal fat, but whether this fat depot is a good representation of visceral fat still remains controversial (Betz et al., 2013). In the study of human patients with pheochromocytoma disease who had performed both ^18^F-fluorodeoxyglucose positron emission tomography/computed tomography (^18^F-FDG PET/CT) and plasma total metanephrine (TMN) measurements in China, browning of human visceral fat has been observed and reduces whole body fat mass by burning more fats through increased UCP1 in beige cells or BAT (Wang et al., 2011). Additionally, recent studies suggest that browning can increase the basal metabolism by burning fat through UCP1 and has been proposed as a potential approach for reducing body fat or treating obesity (Wu et al., 2012; Cummins et al., 2014).

### Autophagy Types in Adipocytes

Adipocytes undergo three major types of autophagy: macroautophagy, macrolipophagy (generally referred as lipophagy), and mitophagy. These occur dynamically depending on browning status (Baerga et al., 2009; Singh et al., 2009b; Zhang et al., 2009; Singh and Cuervo, 2012; Li and Ding, 2017; Ghosh et al., 2018). Both autophagy malfunction and adipocyte dysfunction are clearly connected with the causes of metabolic disorders such as obesity and diabetes (Baerga et al., 2009; Singh et al., 2009b; Zhang et al., 2009; Bjorndal et al., 2011; Singh and Cuervo, 2012; Bluher, 2013; Scheele and Nielsen, 2017; Ghosh et al., 2018). To examine this, both mechanistic and clinical studies have investigated the significant relationship between autophagy and browning (Armani et al., 2014; Stienstra et al., 2014). Here, we summarize the following: 1) the three major types of autophagy and their significance in regulating adipose lipid and energy metabolism; and 2) autophagy manipulations through direct autophagy gene knockdown or chemical/drug administration affecting the browning process in humans and mice from previous publications.

## Lipophagy in Adipocyte Lipid Metabolism

Lipophagy is the selective removal of lipid droplets in cytosolic organelles by lysosomes, which are derived from stimulated autophagy markers such as LC3 and p62 (Singh and Cuervo, 2012; Ward et al., 2016). Lipogenesis, often considered identical to adipogenesis, is focused on the formation of lipid droplets during white adipocyte differentiation with autophagy activation; lipolysis, on the other hand, is the secretion of glycerol and fatty acid partially resulting from the degradation of lipid droplets by autophagy (Cingolani and Czaja, 2016; Ahmed et al., 2018). The balance between lipogenesis and lipolysis plays a vital role in regulating the lipid metabolism in white and brown adipocytes (Singh and Cuervo, 2012; Martinez-Lopez et al., 2016; Zechner et al., 2017). ULK1 activates lipolysis by activating autophagy in 3T3-L1 adipocytes. However, ULK1 inhibits fatty acid synthesis and uptake and activates fatty acid oxidation in the mitochondria independent of autophagy in adipocytes (Ro et al., 2013). In an *in vivo* study of POMC neurons using C57BL/6 WT mice, lipophagy in BAT and liver was activated by both cold exposure and rapamycin administration via the specific surface protein of lipid droplets, adipose triglyceride lipase (ATGL), and LC3 association (Martinez-Lopez et al., 2016). Although both liver and adipose tissue are important tissues in regulating lipid metabolism (Martinez-Lopez et al., 2016), when lipophagy was blocked in liver-specific ATG7 knockout mice, the lipid droplets accumulated in the liver and showed a steatosis-like phenotype (Singh and Cuervo, 2012; Liu and Czaja, 2013). However, in the case of adipose-specific ATG7 knockout mice, white adipocytes showed more brown adipocyte phenotypes with decreased lipids, increased number of mitochondria and beta oxidation (Singh et al., 2009b; Zhang et al., 2009).

The mechanism underlying different tissue specificity is still unclear (Singh and Cuervo, 2012; Martinez-Lopez et al., 2016). When basal lipophagy is inhibited by hyperactivation of mTORC1 due to overnutrition in the human body, lipid droplets are rapidly accumulated in BAT and liver (Singh et al., 2009a). By contrast, when inducible lipophagy is enhanced by inhibition of mTORC1 and activation of AMPK under starvation, lipophagy actively degrades lipid droplets in WAT and releases them as free fatty acids so that other metabolic tissues such as liver and muscle can utilize them as an energy source (Rosen and Spiegelman, 2006; Liu and Czaja, 2013; Ward et al., 2016). Thus, the balance between basal lipophagy and inducible lipophagy, as well as the balance between lipogenesis and lipolysis, is important and seems to be a possible mechanism explaining tissue specificity. BAT and liver tissue would be more prone to the balance between the basal and inducible status of lipophagy, whereas WAT would be more prone to the balance between lipogenesis and lipolysis. These different sensitivities and availability of lipophagy according to the type of tissues and stimuli may create advantages by allowing it to quickly adapt to the different levels of nutrient status in the human body (Martinez-Lopez et al., 2016; Ward et al., 2016). In future studies, transgenic mice with an inducible lipophagy system may serve as a very plausible model for identifying lipophagy specificity and its effect on lipid contents depending on nutrient availability (Singh and Cuervo, 2012).

## Mitophagy in Adipocyte Mitochondria Function

Mitophagy is the process of actively removing excess mitochondria through selective autophagy when mitochondria have accumulated during differentiation or have been damaged by oxidative stress such as ROS (Zhang et al., 2012; Ashrafi and Schwarz, 2013; Li et al., 2015; Taylor and Gottlieb, 2017). Mitophagy can be induced by ULK1 upon AMPK activation or mTORC1 inhibition under cellular maturation or nutrient deprivation (Kundu et al., 2008; Egan et al., 2011; Kim et al., 2011). The main mitophagy process, the association between mitochondria and autophagolysosomes, is mediated by the ubiquitin-dependent PINK1-Parkin pathway (Narendra et al., 2010; Vincow et al., 2013; Bingol and Sheng, 2016). Alternatively, mitochondria can be degraded by selective autophagy via LC3 and p62 protein independent of ubiquitin in adipose tissue (Altshuler-Keylin and Kajimura, 2017; Taylor and Gottlieb, 2017; Lu et al., 2018). Mitochondria can also be degraded and decreased in number through mitophagy to form more lipid droplets in white adipocyte tissue during differentiation by limiting fatty acid oxidation (Gospodarska et al., 2015; Altshuler-Keylin and Kajimura, 2017). Mitophagy at least in part contributes to whitening of beige adipocytes, turning them into white adipocytes by removing mitochondria after the withdrawal of cold exposure (Altshuler-Keylin et al., 2016; Altshuler-Keylin and Kajimura, 2017; Lu et al., 2018). Therefore, when mitophagy is blocked in white adipocytes, mitochondria cannot be degraded and accumulated while inhibiting adipogenesis, which results in a beige/brown adipocyte phenotype (Altshuler-Keylin and Kajimura, 2017). Consistent with cell culture studies, when mitophagy is inhibited in mice either by autophagy gene deficiency or chemical administration, WAT shows accumulation of mitochondria with decreased fat mass and changes into a phenotype like the beige or brown adipocytes (Singh and Cuervo, 2012; Altshuler-Keylin et al., 2016; Taylor and Gottlieb, 2017; Lu et al., 2018). Clinical researchers have observed more accumulation of dysfunctional or metabolically impaired mitochondria in obese people compared to a lean control group (Kraunsoe et al., 2010; Chattopadhyay et al., 2015). These observations possibly suggest that mitophagy would be negatively regulated by excessive fat accumulation or in obese condition. In conditions of overnutrition, mTORC1 activation and mitophagy inhibition resulted in greater accumulation of impaired mitochondria (Altshuler-Keylin and Kajimura, 2017). Studies using autophagy-related gene knockout mice fed with a high-fat diet suggest that when autophagy and mitophagy in adipocytes are impaired by overnutrition, inhibition of lipogenesis and activation of lipophagy can occur as a compensatory mechanism (Zhang et al., 2009; Altshuler-Keylin and Kajimura, 2017). To our surprise, the browning of WAT was observed in skeletal muscle-specific Atg7 knockout mice that were resistant to obesity induced by a high-fat diet (Kim et al., 2013). This ambivalence of mitophagy in adipocyte turnover and the existence of compensation mechanisms with other selective autophagy may be for purposes of more effectively maintaining mitochondrial integrity and mass in adipocytes (Lu et al., 2018). Although mitophagy is suggested as a positive regulator of white adipogenesis and a negative regulator of beige and brown adipogenesis, the level of mitophagy necessary for browning seems controversial due to the complicated regulation of activity dependent on nutrition status (Altshuler-Keylin and Kajimura, 2017). Therefore, the proper modulation of mitophagy in adipocytes in humans and mice seems necessary for the timely turnover between white, beige, and brown adipocytes, dependent on nutrition level.

## Autophagy Manipulations in Adipocyte Browning

Although the distribution of adipose tissue is distinct in humans and mice, both share common characteristics (Seale et al., 2009; Zhang et al., 2018). Anatomically in male mice, interscapular brown adipose tissue (iBAT) contains classic brown adipocytes, whereas epididymal white adipose tissue (eWAT) and subcutaneous white adipose tissue (sWAT) contain classic white adipocytes (Sanchez-Gurmaches and Guertin, 2014; Gospodarska et al., 2015). In humans, most classic brown adipocytes develop mainly around the neck and supraclavicular area through infancy, but gradually decrease until adulthood (Wu et al., 2012; Zhang et al., 2018). Several studies using positron emission tomography (PET)-CT have demonstrated that, in addition to size reduction in aging, BAT activity is reduced in obese and diabetic patients (Lee et al., 2010; Leitner et al., 2017). A few other clinical research groups have suggested that stimulating browning in WAT would be beneficial in slowing obesity, diabetes, and even the aging process (Scheele and Nielsen, 2017). Therefore, the existence of active turnover from WAT to beige fat to BAT in humans and mice has been recognized as a potential therapeutic target for prevention and treatment of obesity and related metabolic diseases (Kajimura et al., 2015; Schrauwen et al., 2015; Giordano et al., 2016). Even whole tissue switching of WAT to BAT through surgical transplantation or implantation of mesenchymal stem cells, brown adipocytes, or BAT into WAT areas in humans and mice is gaining a new spotlight as a novel method to prevent or treat obesity and diabetes (Liu et al., 2013; Soler-Vazquez et al., 2018). However, the activity and selectivity of autophagy after the transplantation or implantation still needs further investigation.

Indeed, autophagy plays an important role in the browning of WAT and beige adipocytes. A recent study has reported that autophagy is needed to convert beige adipocytes to WAT upon removal of β3-AR agonists or recovery from cold exposure (Altshuler-Keylin et al., 2016). Cairo et al. (2016) reported that thermogenic activation through cold exposure inhibits autophagy, which leads to increased UCP1 level in BAT. Although we have selected only a few significant factors to discuss in our review, numerous factors are involved in the manipulation of the autophagy pathway, which regulate the browning of WAT and beige adipocyte (Table 1).

**Table 1:** Summary of recent studies about the effect of direct autophagy gene manipulation or autophagy-related regulators on adipocyte browning.

Study	Target Gene Intervention	Application	Results	Clinical/Physiological Function
Baerga et al., 2009; Singh et al., 2009b	ATG5	*In vitro* Chloroquine (autophagy inhibitor) treatment in MEF cells (Baerga et al., 2009) siAtg5 (knockdown) in MEF cells (Singh et al., 2009b) *In vivo* Histological analysis of Atg5^-/-^ late-stage embryos and neonatal pups (Baerga et al., 2009)	Chloroquine treatment and Atg5 knockdown decreased adipogenesis of MEF cells (Singh et al., 2009b). Perilipin A positive adipocytes in sWAT as dramatically reduced in Atg5^-/-^ late-stage embryos and neonatal pups (Baerga et al., 2009). Chloroquine’s or Atg5’s effect on browning is not observed.	Chloroquine increased success in an autophagy-inhibitor based treatment therapy for a variety of cancer types in humans, when compared to chemotherapy or radiation alone (Xu et al., 2018).
Singh et al., 2009b; Zhang et al., 2009	ATG7	*In vitro* siAtg7 in 3T3-L1 adipocytes (Singh et al., 2009b) *In vivo* Adipose-specific Atg7^-/-^ and WT mice (both groups)	White adipocyte differentiation is blocked upon Atg7 loss, and mice showed brown adipocyte phenotypes with decreased lipids, increased number of mitochondria and beta oxidation.	N/A
Ro et al., 2013	ATG1	*In vitro* Rapamycin treatment, siULK1 or siULK2 in 3T3-L1 adipocytes	ULK1 or ULK2 is necessary for autophagy induction in adipocytes. ULK1 negatively regulates lipogenesis independent of autophagy in adipocytes. ULK1’s or ULK2’s effect on browning is not investigated.	Rapamycin, a potent ATG1 activator through mTORC1 inhibition, has been shown to increase the rate of autophagy in ischemic adipose derived stem cells used during transplantation in humans, promoting overall success of the surgical implantation (Li et al., 2017).
Martinez-Lopez et al., 2016	ATGL	*In vivo* Cold or rapamycin administration on C57BL/6 WT mice	Both cold and rapamycin administration in POMC neuron activates lipophagy in BAT via ATGL-LC3 association.	See above
Cairo et al., 2016; Altshuler-Keylin et al., 2016	UCP1	*In vivo* Cold administration on C57BL/6 WT mice (Cairo et al., 2016) Cold or β3-AR agonist administration on [UCP1(+)]-adipocyte-specific Atg5 or Atg12 knockout mice (Altshuler-Keylin et al., 2016)	Activation of UCP1 suppresses autophagy in BAT (Cairo et al., 2016). Atg5 or Atg12 knockout in beige/brown adipose tissue prevents white-like characteristics through inhibiting autophagic clearance of mitochondria (Altshuler-Keylin et al., 2016).	N/A
Taylor and Gottlieb, 2017; Lu et al., 2018	PARKIN or PARK2	*In vitro* Rosiglitazone (browning stimuli), siParkin, or mCherry-Parkin (overexpression) in 3T3-L1 adipocytes (Taylor and Gottlieb, 2017) *In vivo* Parkin^-/-^ and WT mice with rosiglitazone or CL316243 injection (Taylor and Gottlieb, 2017) *In vivo* Park2 knockout mice administered with β3-AR agonist, CL316243 (Lu et al., 2018)	Rosiglitazone induces browning in 3T3-L1 white adipocytes; Parkin knockdown does not affect browning, but Parkin overexpression inhibits browning in adipocytes (Taylor and Gottlieb, 2017). When browning stimulus CL316243 was removed, UCP1 was reduced in WAT of WT, but was maintained in Park2 knockout mice and BAT of both groups (Lu et al., 2018).	Rosiglitazone, a PPAR-γ agonist, has been shown to increase overall body fat content in humans, but does not affect heart rate variability (Grenier et al., 2016). CL 316,243 has been shown to increase the effectiveness of insulin, and fat oxidation in lean male subjects, by acting as an agonist for β_3_-adrenergic receptor (Weyer et al., 1998).
Armani et al., 2014	Mineralocorticoid receptor antagonist: spironolactone (spiro) or drospirenone (DRSP)	*In vitro* 3T3-L1 was differentiated with 10^-8^ M aldosterone, 10^-5^ M DRSP or 10^-5^ M spiro treatment *In vivo* High-fat diet-fed mice with	white adipocytes, revealed by increase of brown adipose-specific markers such as UCP1 and PRDM16.	Serum aldosterone reduction through diet change and increase in physical activity has been shown to decrease obesity-related health factors in young adults only diagnosed with excess body fat (Cooper et al., 2013).
		6 mg/kg/day DRSP or 20 mg/kg/day spiro for 90 days	Mineralocorticoid receptor antagonists reduced body weight gain and WAT mass gain via autophagy activation, but significantly increased browning of WAT and primary	DRSP, an autophagy activator, currently has no human clinical trials available. Spiro was shown to rescue insulin resistance in humans with chronic kidney disease, and reversed glucose intolerance in mice possibly by activating autophagy (Hosoya et al., 2015).
Ghosh et al., 2018	PIK3C3	*In vivo* Aged C57BL/6 male mice with mutant PIK3C3 gene	PIK3C3 mutation led to enhanced browning of gWAT when autophagy is impaired.	N/A
Parray and Yun, 2017	Thiodigalactoside (TDG)	*In vitro* 500 μM during 3T3-L1 and HIB1B adipocyte differentiation *In vivo* 5-week-old rats peritoneal injected 5 mg/kg/week for 5 weeks	TDG, an inhibitor of autophagy, increases browning markers, thermogenic protein UCP1, and mitochondrial functions and activities.	TDG currently has no clinical data available in humans.
Leu et al., 2018	Raspberry ketone	*In vitro* 100 μM for 48 hrs after fully differentiated 3T3-L1 adipocytes *In vivo* 8-week-old rats administered 160 mg/kg/day for 8 weeks	Raspberry ketone-fed rats had less adipose tissue, more browning-related markers through inhibition of autophagy.	Currently, clinical data is not available in humans for Raspberry ketone.

### Parkin-Mediated Mitophagy in Browning

Parkin (gene name: Park2) is a E3 ubiquitin ligase that plays a critical role in ubiquitination as a mitophagy-associated degradation signal (Geisler et al., 2010; Jin and Youle, 2012; Pickrell and Youle, 2015). The role of Parkin in browning of WAT has been studied in 3T3-L1 adipocytes and the Parkin-deficient C57BL/6 mice model. Parkin expression increases during 3T3-L1 adipocyte differentiation, while its expression decreases in rosiglitazone-treated 3T3-L1 adipocytes, which have phenotypes of beige adipocytes due to enhanced UCP1 expression. Inhibition of the Parkin gene does not affect browning, but overexpression of Parkin significantly reduces browning in adipocytes (Taylor and Gottlieb, 2017). Furthermore, Parkin is highly expressed during beige adipocyte differentiation (Lu et al., 2018). The Kajimura group has shown that Parkin is required to maintain beige adipocytes in WAT. When CL316243, a β3-AR agonist, is removed, UCP1 expression is significantly reduced in WAT of wild type (WT) mice, but still expressed in WAT of Park2 knockout mice. In contrast, UCP1 expression in BAT is not changed in both WT and Park2 knockout mice after CL316243 is removed (Lu et al., 2018).

### Mineralocorticoid Receptor Antagonism in Browning

The Yan group has shown that autophagy is regulated by mineralocorticoid receptor (MR) antagonism (Li et al., 2016). Spironolacton induces LC3 and ATG5 expression and reduces PI3K/AKT/mTOR pathways in injured human podocytes (Li et al., 2016). Previous research has reported the role of MR in adipocyte differentiation. Drospirenone (DRSP) significantly reduces 3T3-L1 and 3T3-F442A adipocyte differentiation without cell cytotoxicity (Caprio et al., 2011). MR also regulates browning of WAT though autophagy. Additionally, it has been determined that MR antagonists fully prevent aldosterone-induced autophagy in white adipocytes along with an increase of UCP1 expression. MR antagonists significantly enhance browning of WAT in diet-induced obese mice as well as brown adipose-specific markers in primary adipocytes isolated from WAT (Armani et al., 2014).

### PIK3C3 in Browning

PIK3C3 is a subunit of class III phosphoinositide 3-kindase (PI3K) that phosphorylates phosphatidylinositol to generate phosphatidylinositol 3-phosphate. The PIK3C3-ATG14 complex induces autophagy especially in nutrient-stress conditions such as starvation (Yuan et al., 2013). Recently, it has been shown that in aged mice with a PIK3C3 mutation, compared to fl/fl control mice, adipogenesis markers, such as AP2 and C/EBP-α, are reduced, but brown adipose-specific markers, such as UCP1 and PPAR- γ coactivator (PGC)1α, are enhanced in both mRNA and protein levels in the gonadal WAT (gWAT), possibly through blocking of autophagy (Ghosh et al., 2018).

### Thiodigalactoside in Browning

Thiodigalactoside (TDG), an inhibitor of galectin 1 and autophagy, has recently been studied in obesity research by the Yun group (Mukherjee et al., 2015; Parray and Yun, 2015; 2017). They have reported that TDG-treated adipocytes significantly inhibit lipid accumulation, and TDG also reduces body weight in high-fat diet-fed rats (Mukherjee et al., 2015). Their second study has shown proteomic identification of TDG in WAT of rats with high-fat diet-induced obesity. Specifically, proteins involved in carbohydrate metabolism and the tricarboxylic acid cycle remarkably increased in WAT of TDG-injected obese rats (Parray and Yun, 2015). A most recent study has reported that TDG plays an important role in browning of white adipocytes and WAT in obese rats (Parray and Yun, 2017). Dose-dependent TDG treatment reduces galectin 1 and ATG 5 gene expression, but enhances brown-specific markers, UCP1 and PGC1α, in 3T3-L1 adipocytes. Moreover, UCP1 and PGC1α gene and protein expressions are upregulated by PDG injection in iWAT, eWAT, and BAT of diet-induced obese rats, possibly through inhibition of ATG5/LC3-II and increase of p62 expression (Parray and Yun, 2017).

### Raspberry Ketone in Browning

Raspberry ketone, 4-(4-hydroxyphenyl) butan-2-one, a phenolic compound found in red raspberry, has emerged as a dietary bioactive compound with beneficial effects on obesity (Cotten et al., 2017; Tsai et al., 2017). Evidence suggests that raspberry ketone reduces body weight and food intake in high-fat diet-fed mice (Cotten et al., 2017) and ovariectomy-induced obese rats (Leu et al., 2017). In addition, raspberry ketone inhibits 3T3-L1 adipogenesis, revealed by inhibition of expression of adipogenesis markers such as PPAR-γ, C/EBP-α, FAS, and AP2, possibly via inhibition of autophagy, confirmed by decrease of ATG12 and LC3B levels, as well as increase of p62 and mTORC1 levels (Leu et al., 2017; Tsai et al., 2017). It has been recently reported that high concentration of raspberry ketone (100 μM) significantly increases browning of 3T3-L1 adipocytes, revealed by an increase of browning-specific markers, including UCP1 and PGC1α, and lipolysis markers such as hormone-sensitive lipase and triglyceride lipase (Leu et al., 2018). Moreover, expression of brown adipose markers is increased in ovariectomy-induced obese rats that have been administered raspberry, compared to control groups mediated by inhibition of ATG12 and an increase of p62 expression (Leu et al., 2018).

## Conclusion: Autophagy- and Browning-Targeting Therapeutics for the Prevention of Obesity

**FIGURE 1:**
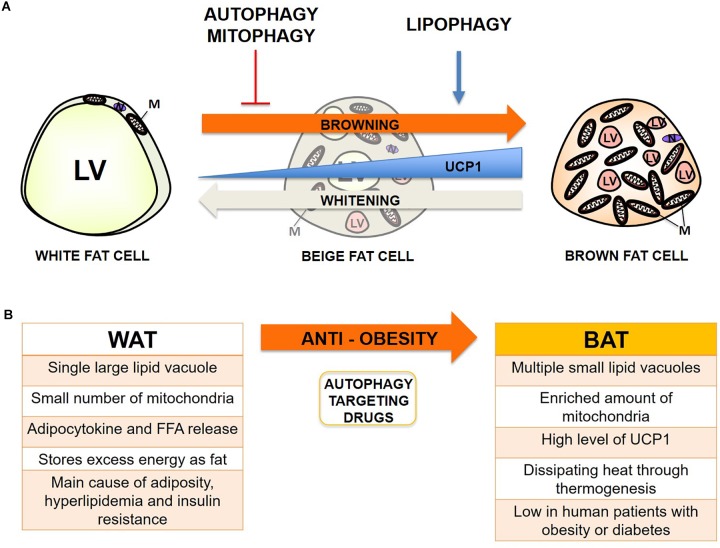
Autophagy effect on adipocyte browning and potential therapeutic target for prevention of obesity. **(A)** Summary of the proposed effect of autophagy, lipophagy, and mitophagy on adipocyte browning from recent studies. LV, lipid vacuoles; M, mitochondria; N, Nucleus. **(B)** Summary of the main characteristics of WAT and BAT in obesity, and the induction of browning through manipulation of autophagy as a promising target for anti-obesity therapy.

Adipose metabolism is closely linked with metabolic dysfunctions such as obesity and diabetes when fat distribution and energy balance through mitochondria are not strictly maintained (Bjorndal et al., 2011; Bluher, 2013; Stienstra et al., 2014; Scheele and Nielsen, 2017). The relationship between adipose metabolism and autophagy has become an increasingly intriguing topic since the dawn of the discovery of selective autophagy, including lipophagy and mitophagy, which can also actively occur in adipose tissue (Rocchi and He, 2015). Autophagy has previously been shown to be increased in adipocytes from obese humans and mice (Ost et al., 2010; Kovsan et al., 2011; Jansen et al., 2012). Previous reports indicate that excess free fatty acid (FFA) – particularly saturated FFA like palmitic acid (PA), but not unsaturated FFA such as oleic acid (OA) – formed by a high-fat diet can activate autophagy through JNK2 or PKC activation (Tan et al., 2012; Tu et al., 2014). Conversely, lipophagy seems beneficial for degrading excess fats from WAT and generating more intracellular space for the expansion of the mitochondrial contents from BAT when mitophagy is inhibited or mitochondrial biogenesis is activated, thus protecting the human body from nutrient oversupply which, can occur in obesity conditions (Singh and Cuervo, 2012; Cummins et al., 2014). Mitophagy seems much more controversial because basal activity can be beneficial through elimination of damaged mitochondria from accumulated ROS in obesity; however, hyperactive inducible mitophagy can convert BAT or beige fat to white during differentiation, which is called “reverse browning or whitening,” and subsequently cause systemic change in adipocytokine release and lipid metabolism (Hill et al., 2012; Gospodarska et al., 2015). Overall, inhibition or deficiency of autophagy, activation of lipophagy rather than lipolysis, and a basal or moderate level of mitophagy seems the most optimal combination for the prevention of obesity so far (Figure 1).

However, we have also encountered a few exceptional cases from previous reports noting that hyperactive autophagy can be beneficial during hypermetabolic conditions such as hepatic steatosis, atherosclerosis, injuries from burning, sepsis, and cachexia (Volzke et al., 2005; Penna et al., 2014; Pravda, 2014; Song et al., 2014; Abdullahi and Jeschke, 2016). Normally, hyperactivation of autophagy leads to apoptotic cell death (Lum et al., 2005), but highly autophagic cells under hypermetabolic conditions such as post-thermal injury may survive better by efficiently regulating energy metabolism (Auger et al., 2017). Paradoxically, autophagy activation in WAT can be beneficial for obese or diabetic patients with a hypermetabolic profile or complications, because it decreases FFA and glycerol release from hypertrophic and hyperplasic WAT by actively degrading lipid vacuoles (LV) as an energy source. We have summarized previously reported autophagy and selective autophagy manipulations and their effect on adipocyte browning (Table 1). Since the combined delicate manipulation of autophagy, lipophagy, and mitophagy seems necessary for the timely turnover between white, beige, and brown adipocytes dependent on nutrition levels in humans and mice, the direct manipulation of autophagy and selective autophagy or the administration of autophagy-targeting drugs should be cautiously performed. Finally, summarizing autophagy regulation and its implications in browning could help give insights for the development of autophagy-targeting drugs in the prevention of obesity.

## Author Contributions

All authors listed have made a substantial, direct and intellectual contribution to the work, and approved it for publication.

## Conflict of Interest Statement

The authors declare that the research was conducted in the absence of any commercial or financial relationships that could be construed as a potential conflict of interest.
